# Effects of Acidic Polysaccharide-Enriched Extracts from *Holothuria tubulosa* on Two- and Three-Dimensional Invasive Breast Cancer Cell Models

**DOI:** 10.3390/biology14040334

**Published:** 2025-03-25

**Authors:** Cristina Ciampelli, Sylvia Mangani, Gabriele Nieddu, Marilena Formato, Paraskevi Ioannou, Spyros Kremmydas, Nikos Karamanos, Antonio Junior Lepedda

**Affiliations:** 1 Department of Biomedical Sciences, University of Sassari, Viale San Pietro, 43b, 07100 Sassari, Italy; c.ciampelli@phd.uniss.it (C.C.); ganieddu@uniss.it (G.N.); formato@uniss.it (M.F.); 2 Biochemistry, Biochemical Analysis & Matrix Pathobiology Research Group, Laboratory of Biochemistry, Department of Chemistry, University of Patras, 26504 Patras, Greece; up1111418@upatras.gr (S.M.); ioannouevi@icloud.com (P.I.); spyroskremmydasns@gmail.com (S.K.); n.k.karamanos@upatras.gr (N.K.)

**Keywords:** marine invertebrates, acidic polysaccharides (APs), anticancer properties, breast cancer, epithelial–mesenchymal transition (EMT)

## Abstract

Marine invertebrates, particularly Holothurians, have emerged as valuable sources of bioactive compounds with potential anticancer properties. In this study, we investigated the effects of two acidic polysaccharide-enriched (APs) fractions (Ht1 and Ht2) from the sea cucumber species *Holothuria tubulosa* on the highly invasive cell line MDA-MB-231. Functional assays were performed to assess cell viability, migratory potential, adhesion on collagen I, and cell morphology, alongside gene expression analysis. Additionally, a preliminary evaluation of their effects on three-dimensional breast cancer cell-derived spheroids was conducted. Both AP fractions exerted anticancer effects by decreasing cell viability. Ht1 showed a significant inhibitory effect on cell migration, increased adhesion on collagen I, and exhibited a trend to transform the mesenchymal MDA-MB-231 cells to a more epithelial phenotype. Treatment with the AP fractions modulated the expression of genes, such as the epithelial marker E-cadherin (for the Ht1), a key cell adhesion molecule, and the matrix metalloproteinases 7 and 9 (for the Ht2), enzymes involved in extracellular matrix remodeling, which hold critical roles in cancer progression and metastasis. No significant effects were observed on spheroids, possibly due to the high charge and hydrophilicity of the APs, leading to poor penetration into the inner spheroid layers. Although preliminary, these findings highlight the potential of *H. tubulosa*-derived APs as promising antineoplastic agents, warranting further investigation into their mechanisms of action and structural characterization.

## Simple Summary

Holothurians represent a rich source of bioactive compounds with potential applications in pharmaceutics. We recently described interesting inhibitory properties on both the intrinsic and extrinsic coagulation pathways of the acid polysaccharides component extracted from the body wall of the species *Holothuria tubulosa*. The highly sulphated fractions, mainly consisting of a fucosylated chondroitin sulfate, were the most active. In this study, we assessed the effects of the less sulphated polysaccharide fractions on MDA-MB-231 cells, a highly metastatic triple-negative breast cancer cell line. Specifically, the effects on cell metabolic activity, migratory behaviour, and the expression of key genes implicated in tumor progression were evaluated. Furthermore, a qualitative assessment of the morphology of three-dimensional tumor spheroids was performed. The findings suggest that these fractions may exert antitumor effects by reducing the metabolic activity and migratory potential of MDA-MB-231 cells.

## 1. Introduction

Sea cucumber is an interesting natural source of novel functional materials with biological activities that could be used in food, as well as in biomedicine industries [1,2]. Historically, sea cucumbers have been valued in traditional medicine, particularly in Asia, for their health benefits, including their use as an anti-inflammatory, wound healing, and anticancer agent [3]. In this respect, promising anticancer properties, including cytotoxic activity, induction of apoptosis, cell cycle arrest, inhibition of tumor growth, anti-metastatic and anti-angiogenic activities, and inhibition of drug resistance, have been reported for some of their bioactive compounds [4]. Among them, the fucosylated chondroitin sulfate (FCS) purified from the body wall of sea cucumber exhibited a range of biological effects due to its structural heterogeneity in terms of monosaccharide composition, degree of sulfation, molecular weight, and branching patterns. According to a very recent literature survey, 10 different biological activities of FCS purified from 42 sea cucumber species have been documented so far by 104 publications, including anti-inflammatory, anti-oxidant, anti-angiogenic and anti-coagulant properties [5]. Recently, we described an interesting dose-dependent inhibitory effect on both the intrinsic and extrinsic coagulation pathways of two highly sulfated polysaccharide fractions, mainly consisting of a FCS, extracted from *Holothuria tubulosa* [6]. These polysaccharides were found to be highly concentrated throughout the body wall and cytocompatible, ultimately suggesting their potential suitability in different biomedical applications.

Breast cancer represents the most common malignancy and is the leading cause of cancer-associated mortality in women worldwide [7]. According to the World Health Organization, 2.3 million women were diagnosed with breast cancer and it accounted for 670,000 deaths globally in 2022, highlighting its widespread impact on global health (https://www.who.int/news-room/fact-sheets/detail/breast-cancer, accessed on 1 January 2025). Various risk factors have been identified, including genetic predispositions such as BReast CAncer gene (BRCA) mutations, hormonal influences and lifestyle factors. Age, family history, and environmental factors further contribute to the complex pattern of breast cancer etiology [8]. Breast cancer molecular classification is primarily determined by the expression of specific hormone receptors, including the estrogen receptor (ER), progesterone receptor (PR), and the human epidermal growth factor receptor 2 (HER2). The main breast cancer subtypes include ER-positive/PR-positive, HER2-positive, and triple-negative breast cancer (TNBC), characterized by the absence of ER, PR, and HER2. Each subtype has unique biological behavior and response to therapy, making molecular profiling an essential component of personalized treatment strategies [9]. Hormone receptor-positive breast cancer, representing around 70% of all cases, typically responds well to endocrine therapies that target estrogen signaling. HER2-positive breast cancer, accounting for approximately 15–20% of cases, is the most aggressive among the hormone receptor-positive types, but it can be effectively treated with targeted therapies [10]. In contrast, TNBC, which represents 10–15% of cases, results in more challenging treatment due to the lack of targeted therapies and is often associated with a poorer prognosis [11]. New potential therapeutic targets are constantly being identified to fight this deadly disease [12]. Gaining insight into the underlying molecular pathways is crucial for developing effective treatment strategies for this highly complex and heterogeneous disease.

The MDA-MB-231 cell line is one of the most widely studied models in breast cancer research, particularly in investigations focusing on TNBC. It is characterized by the lack of estrogen (ERα-negative, but ERβ-positive) and progesterone receptors, as well as low levels of HER2 [13]. Additionally, MDA-MB-231 cells can be used to study epithelial–mesenchymal transition (EMT), a critical process associated with increased invasiveness and metastatic potential [14]. Finally, this cell line represents a model for studying the role of the extracellular matrix (ECM) and cell–ECM interactions in cancer cell functional properties, highlighting potential therapeutic strategies for TNBC management [15].

The ECM is increasingly recognized as an important regulator of cancer progression, particularly breast cancer [16]. It plays a pivotal role in both the structural and biochemical regulation of tissues and its dysregulation is closely linked to the onset and progression of breast cancer. ECM is a non-cellular three-dimensional macromolecular network composed of collagens, proteoglycans/glycosaminoglycans, elastin, fibronectin, laminins, and several other glycoproteins [17]. Within the ECM, proteoglycans can shield growth factors from circulating proteases and establish gradients that guide cell migration [18]. In normal mammary tissue, the ECM maintains the tissue architecture and regulates essential processes, including cell adhesion, migration, and differentiation, by inducing downstream signaling pathways. However, during breast cancer development, the composition, stiffness, and organization of the ECM are significantly altered through a process known as ECM remodeling [19].

The ECM is not only a structural entity, but also a dynamic environment that actively regulates cellular behavior. Through its interaction with cell surface receptors, primarily integrins, the ECM influences cell signaling pathways that control a wide variety of cellular functions. This interaction is bidirectional as cells can, vice versa, remodel the ECM by secreting enzymes such as matrix metalloproteinases (MMPs), which degrade ECM components, and by producing new ECM molecules [20,21]. Overexpression of matrix-degrading enzymes by cancer cells results in extensive reorganization of the ECM, further contributing to cell migration and invasion [22]. Extensive research during the last decade has unraveled the significance of ECM remodeling at each stage of metastasis development, from surviving in circulation to forming the pre-metastatic and metastatic niches [23].

Among the various factors contributing to the aggressiveness and metastatic potential of breast cancer, EMT plays a pivotal role. EMT is a complex biological process through which epithelial cells lose their cell polarity and intercellular adhesion, acquiring mesenchymal characteristics that result in increased migratory capacity, enhanced invasiveness, and resistance to apoptosis [21,22]. This transition is critical in physiological processes, such as embryonic development and wound healing, but it is also pathologically activated in cancer, facilitating tumor progression and metastasis [24]. During EMT, breast cancer cells undergo a series of morphological and molecular changes, resulting in the downregulation of epithelial markers, such as E-cadherin, and the upregulation of mesenchymal markers, including N-cadherin, vimentin, and fibronectin [25]. EMT is regulated by a broad range of signaling pathways and transcription factors, such as TGF-β, Wnt, Notch, and Hedgehog, which are frequently dysregulated in breast cancer. These pathways converge on a key group of transcription factors, including Snail, Slug, Twist, and ZEB1/2, which coordinate the EMT process by suppressing epithelial gene expression and promoting the transcription of genes related to mesenchymal phenotype [26]. Recent studies have suggested that a complete EMT is not always necessary for metastasis and that epithelial–mesenchymal hybrid states may be more conducive to metastatic dissemination and colonization [27].

Despite advances in diagnosis and treatment, TNBC remains a leading cause of cancer-related mortality, necessitating continued exploration of novel therapeutic strategies. In this respect, natural compounds could offer more effective and less toxic treatment options. So far, a considerable number of marine natural products have been identified as antineoplastic drugs [28].

In this study, we assessed the effects of two acidic polysaccharide containing fractions, extracted from *Holothuria tubulosa*, on MDA-MB-231 cells, a highly metastatic TNBC cell line. Specifically, their in vitro effects on cell viability, migratory behavior, and morphology, as well as the expression of key genes implicated in tumor progression, were evaluated. While 2D cell cultures offer valuable insights into studying a variety of molecular and functional properties of cells, they lack the dynamic cell–cell interactions found in solid tumors in vivo. Consequently, research is shifting toward the development of advanced three-dimensional cancer cell models, such as spheroids, to better mimic the intricate cell–cell and cell–ECM interactions in vitro.

## 2. Materials and Methods

All reagents and chemicals used in this study were of analytical grade and obtained from Merck Group (KGaA, Darmstadt, Germany), unless otherwise specified.

### 2.1. Specimen Collection

*Holothuria tubulosa* (Echinodermata, Holothuroidea, Holothuriidae) is a benthic, sedentary, complex Metazoa living in the Mediterranean Sea (surface to 100 m depth). Its bilateral body is roughly cylindrical along the oro–aboral axis and dark brown, with numerous conical papillae. The body wall of connective tissue, rich in ECM, with embedded calcareous skeletal ossicles, encircles the coelom in which internal organs are localized. Wild specimens of *H. tubulosa* were collected along the Porto Torres coast (40°50′17.781″ N 8°24′33.955″ E, Asinara Gulf, Northern Sardinian Sea, Western Mediterranean). The animal study protocol was approved by Regione Autonoma della Sardegna (RAS AOO 06-01-00 Autor. Pesca Scient n.13 Prot. Uscita n. 26518 del 25 October 2024).

### 2.2. Acidic Polysaccharides Enrichment

Extraction and fractionation of acidic polysaccharides (APs) was conducted as previously described [6]. Briefly, body wall fragments of *H. tubulosa* were put in absolute ethanol (20 mL per gram of tissue) for 120 h at 4 °C and then finely minced. Tissue dehydration and delipidation were carried out by incubating in acetone (20 mL per gram of tissue) for 24 h. After complete acetone evaporation (about 72 h at 60 °C), the dehydrated and delipidated tissue (DDT) was rehydrated with 0.1 M sodium acetate buffer, pH 6.0, containing 5 mM EDTA and 5 mM cysteine (15 mL per gram of tissue), at 4 °C for 24 h. Proteolytic treatment was performed at 56 °C for 48 h by adding papain (Thermo Fisher Scientific, Waltham, MA, USA, cod. 416761000) (1 U per mg of DDT) to the digestion buffer. Digestion was stopped at 100 °C for 5 min and, after centrifugation at 5000× *g* for 10 min, the supernatant was recovered and immediately loaded onto a chromatography column packed with DEAE-Sephacel anion-exchange resin (Cytiva, Marlborough, MA, USA, cod. 17-0500-01) (10 mL of resin per gram of digested tissue), previously equilibrated with 50 mM sodium acetate, pH 6.0. APs were fractionated by performing two elution steps using 20 mM Tris–HCl buffer, pH 8.6, containing, sequentially, 0.5 M and 1 M lithium chloride. Finally, the eluates were concentrated and dialyzed against deionized water using Amicon Ultra-15 Centrifugal Filter Units with a 3 kDa cut-off according to the manufacturer’s instructions (Millipore, Burlington, MA, USA, cod. UFC9003). The following acronyms have been assigned to each purified fraction to indicate the different elutions: Ht1 for the 0.5 M fraction and Ht2 for the 1 M fraction.

#### 2.2.1. Hexuronic Acid Quantification

Each eluate was assayed for uronic acid (UA) content using the method of Bitter and Muir [29] with glucuronolactone as the standard, as previously reported [30]. Briefly, 250 µL of either standard (from 5 to 40 µg UA/mL) or eluate were added with 1.25 mL of 25 mM sodium tetraborate decahydrate in concentrated sulfuric acid and incubated at 85 °C for 10 min. Afterward, 50 µL of carbazole was added, and, following 15 min of boiling, the absorbance was read at 530 nm. All samples were assayed in duplicate.

#### 2.2.2. Carbohydrate Polyacrylamide Gel Electrophoresis (C-PAGE)

Carbohydrate electrophoresis was carried out in a Mini Protean II cell vertical slab gel electrophoresis apparatus, using 13.5% T, 3% C polyacrylamide running gels, overlaid with a 5% T, 3% C stacking gel, using 40 mM acetic acid and 40 mM Tris–HCl solution, pH 7.8, as the running buffer. A volume corresponding to 2.5 µg, in terms of UA, of each standard polysaccharide and AP fraction was freeze-dried and then resolubilized in 10 µL of 62.5 mM Tris–HCl, pH 7.8, 10% glycerol, and 0.002% cresol red and loaded into the wells. The run was performed at 50 V for the first 10 min and then at 120 V until the dye front reached the bottom of the gel. Staining was performed by incubating the gel with 50 mL of a 50% ethanol solution containing 0.005% Stains-all dye (Merck, KGaA, Darmstadt, Germany, cod. E9379) overnight and in the dark at room temperature. Gels were rehydrated with distilled water before being photographed with a Nikon (Minato, Tokyo, Japan) D3100 reflex camera and acquired using a GS-800 calibrated densitometer (Bio-Rad, Hercules, CA, USA). Electrophoretic profiles were compared with standard polysaccharides: high-molecular-weight hyaluronic acid (HMW-HA) sodium salt from *Streptococcus equi* (Merck, KGaA, Darmstadt, Germany, cod. 63357), low-molecular-weight hyaluronic acid (LMW-HA) sodium salt from *Streptococcus equi* (Merck, KGaA, Darmstadt, Germany, cod. 40583), fucoidan from marine brown algae *Macrocystis pyrifera* (Merck, KGaA, Darmstadt, Germany, cod. F8065), chondroitin sulfate (CS) sodium salt from bovine trachea (Merck, KGaA, Darmstadt, Germany, cod. C9819), and heparin (Hep) sodium salt from porcine intestinal mucosa (Merck, KGaA, Darmstadt, Germany, cod. H3393).

### 2.3. Cell Cultures

The MDA-MB-231 breast cancer cell line was obtained from the American Type Culture Collection (ATCC, Manassas, VA, USA). The cells were cultured at 37 °C in a humidified atmosphere of 5% CO_2_. Dulbecco’s Modified Eagle Medium (DMEM, LM-D1110/500, Biosera, Nuaillé, France) was used as cell culture medium, supplemented with 10% fetal bovine serum (FBS, FB-1000/500, Biosera, Nuaillé, France), antimicrobial agents (100 IU/mL penicillin, 100 µg/mL streptomycin, 10 µg/mL gentamycin sulfate and 2.5 µg/mL amphotericin B), 1 mM sodium pyruvate, and 2 mM L-glutamine (Biosera, Nuaillé, France). The cells were harvested using trypsin–EDTA in PBS (LM-T1706/500, Biosera, Nuaillé, France). All experiments were conducted in serum-free conditions using three biological replicates.

#### 2.3.1. Cell Viability Assay

MDA-MB-231 cells were seeded on 96-well plates at a density of 5000 cells per well. After 24 h incubation in complete cell culture medium, the cells were serum starved overnight. Ht1 and Ht2 were added to the culture medium at a range of concentrations between 0.25–2.5 µg/mL and 5–10 µg/mL, respectively, and cell cultures, including the untreated controls, were incubated for additional 24 h. Water-soluble tetrazolium salt (WST-1; Merck, KGaA, Darmstadt, Germany, cod. 5015944001) assays were performed to evaluate the effects on breast cancer cell viability. The tetrazolium salt produces a highly water-soluble formazan by mitochondrial dehydrogenases in the presence of an intermediate electron acceptor, such as 1-methoxy PMS. The amount of formazan produced is directly proportional to the amount of mitochondrial dehydrogenases in the cell culture [31]. Briefly, after the treatment period, 10 µL of WST-1 reagent was added directly to each well. The plate was incubated with WST-1 for 1 h at 37 °C, protected from light. Finally, the absorbance was measured at 450 nm, using a TECAN microplate reader (TECAN Life Sciences, Männedorf, canton of Zürich, Switzerland), with a reference wavelength between 620–650 nm. The obtained dose–response curves were analyzed to calculate the IC_50_ for both Ht1 and Ht2.

#### 2.3.2. Wound Closure Assay

MDA-MB-231 cells were seeded on 48-well plates at a density of 1 × 10^4^ cells per well. The cells were cultured in complete cell culture medium for 24 h and were serum starved overnight. The next day, the cell monolayer was wounded by scratching with a sterile 10 µL pipette tip. The wells were then washed twice with PBS to remove the detached cells. To minimize the possible contribution of cell proliferation to the migration results, the assay was performed by using a serum-free medium containing the cytostatic cytarabine (10 µM). After a 40 min incubation, serum was added with either Ht1 1.5 µg/mL or Ht2 5 µg/mL (final concentrations) and the wound closure was captured at 0 h, 24 h, and 48 h time points using a digital camera connected to a phase-contrast microscope and quantified by using Fiji v1.52p image analysis software (a distribution of ImageJ). The wound closure in the presence of Ht1 or Ht2 in the culture medium was calculated as the ratio between the wound area difference t0 − t1 or t0 − t2 (24 h or 48 h, respectively for t1 and t2) and t0 wound area, normalized for controls.

#### 2.3.3. Collagen Type I Cell Adhesion Assay

MDA-MB-231 cells were seeded on 6-well plates at a density of 2.5 × 10^5^ cells per well. The cells were cultured in a complete cell culture medium for 24 h and were serum starved overnight. The next day, the Ht1 and Ht2 fractions were added at a final concentration of 1.5 μg/mL and 5 μg/mL, respectively, in a serum-free medium. Meanwhile, 96-well plates were prepared with 40 µg/mL collagen type I (Merck, KGaA, Darmstadt, Germany, cod. C9791) in PBS and were stored at 4 °C overnight. The following day, the plates were washed twice with PBS and incubated in a blocking solution with 2% BSA in PBS for 30 min. After a 24 h incubation, the cells in the 6-well plates were harvested, centrifuged and resuspended in a serum-free medium containing 0.1% BSA. The cells were then seeded in the pre-coated collagen type I 96-well plates at a density of 1 × 10^4^ cells per well. After seeding, the cells were incubated at 37 °C for 40 min to allow for adherence to the collagen type I. Subsequently, the cells were washed twice with PBS to remove the non-adherent cells. To determine the adhesion rate, the reduction potential, positively correlated with cell viability, was evaluated using PrestoBlue™ reagent (Thermo Fisher Scientific, Waltham, MA, USA, cod. A13261). Following 10 min incubation, the emission at 615 nm wavelength was assessed (excitation wavelength 535 nm) using a Victor X5 microplate reader (Perkin Elmer, Waltham, MA, USA).

#### 2.3.4. Immunofluorescence Staining and Microscopy

MDA-MB-231 cells were seeded on glass coverslips in 24-well plates at a density of 5 × 10^4^ cells per well. The cells were cultured in complete cell culture medium for 24 h and then serum starved overnight. The next day, Ht1 and Ht2 fractions were added at a final concentration of 1.5 μg/mL and 5 μg/mL, respectively, and the cells were incubated for 24 h. Following a PBS washing, the cells were fixed in 4% paraformaldehyde for 30 min. Afterward, the cells on the coverslips were washed three times with PBS containing 0.05% Tween 20 and were permeabilized with PBS containing 0.2% Triton X-100. Samples were blocked with PBS containing 5% BSA and 0.05% Tween 20 (blocking solution) for 30 min, followed by incubation with the same solution containing an anti-β-actin primary antibody (Cell Signaling Technology, Danvers, MA, USA, #4970, 1:500) overnight at 4 °C. Afterward, samples were incubated with the blocking solution containing the secondary antibody Alexa Fluor^®^ 546 (Thermo Fisher Scientific, Waltham, MA, USA, 1:1000) for 1 h in the dark, and the glass coverslips were mounted on slides with DAPI in Mowiol mounting medium and the fluorescence was revealed with a Leica TCS SP5 confocal microscope with LAS AF lite 4.0 image software (Leica Microsystems, Wetzlar, Germany).

### 2.4. RNA Isolation, cDNA Synthesis and Real-Time PCR

MDA-MB-231 cells were cultured in Petri dishes at a density of 60 × 10^4^ cells for 24 h and were serum starved overnight. The fractions were added at final concentrations of 1.5 µg/mL for Ht1 and 5 µg/mL for Ht2 and, after 24 h incubation, the cells were collected, and RNA isolation was carried out using the NucleoSpin^®^ RNA II Kit (Macherey-Nagel, Allentown, PA, USA). To quantify the isolated RNA, the absorbance of each sample was measured at 260 nm and RNA purity was determined by evaluating the 260/280 nm and 260/230 nm ratios. For the cDNA synthesis, the PrimeScript™ 1st strand cDNA synthesis kit Perfect Real Time (Takara Bio Inc., Kusatsu, Japan) was used. Real-time PCR was conducted using KAPA Taq ReadyMix DNA Polymerase (KAPA BIOSYSTEMS, Wilmington, MA, USA) according to the manufacturer’s instructions in a 20 µL reaction mixture. The amplification was performed utilizing Rotor Gene Q (Qiagen, Hilden, Germany). The genes of interest and the corresponding primers used are reported in Table 1. Annealing temperatures were set at 60 °C. All the reactions were performed in duplicate. To provide quantification, the point of product accumulation in the early logarithmic phase of the amplification plot was defined by assigning a fluorescence threshold above the background, defined as the threshold cycle (Ct) number. Relative expression of different gene transcripts was calculated using the ∆∆Ct method. The Ct of any gene of interest was normalized to the Ct of the normalizer (Actin). Fold changes (arbitrary units) were determined as 2^−∆∆Ct^.

### 2.5. MDA-MB-231 Spheroid Formation and Treatment

For the 3D cultures, the cells were grown at a density of 10^4^ cells per well in U-shaped round bottom ultra-low adhesion 96-well plates, allowing for spheroid formation after cell self-aggregation (SPL Life Sciences, Pocheon-si, Republic of Korea). After 72 h incubation in a complete cell culture medium, the cells were serum starved overnight. Ht1 and Ht2 were added at final concentrations of 1.5 µg/mL and 5 µg/mL, respectively, and the spheroids’ morphology was captured at 24 h of treatment, using a digital camera connected to a phase-contrast microscope. Each experimental condition was assayed in quadruplicate.

### 2.6. Statistical Analysis

Student’s *t*-test was performed to evaluate the effects of Ht1 and Ht2 on viability, migratory potential, and adhesion of MDA-MB-231 cell cultures. Data are expressed as means ± standard deviations of experiments conducted in triplicate. Differences were considered statistically significant for a *p* value < 0.05. Statistical analysis and graphs were conducted using GraphPad Prism 8 (GraphPad Software, San Diego, CA, USA).

## 3. Results

### 3.1. Electrophoretic Profiles of Holothuria tubulosa-Derived APs

Preliminary structural characterization of hexuronate-containing AP fractions was performed through comparative analysis of electrophoretic profiles on a polyacrylamide gel. Carbohydrate electrophoresis (C-PAGE) allows for the separation of polysaccharides based on their molecular weights. It is worth noting that the treatment with the cationic carbocyanine dye stains all polysaccharides with specific colors. Notably the color and its density depend on the sulfation degree of the polysaccharides, turning from blue to purple to yellow as the degree of sulfation increases.

**Figure 1:**
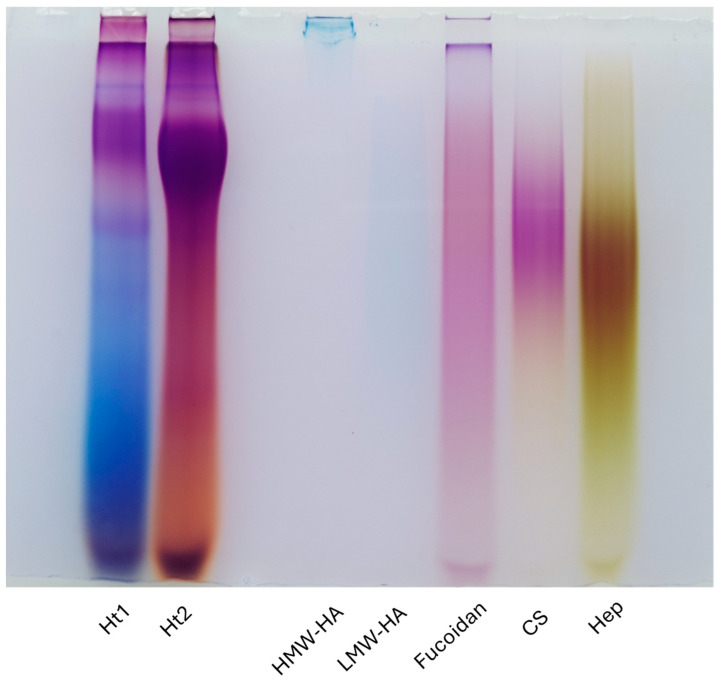
Electrophoretic patterns obtained by C-PAGE and Stains-all dye staining. Ht1 and Ht2 fractions were separated on 13.5% T, 3% C polyacrylamide gels and revealed by the cationic carbocyanine dye Stains-all. The different colors, due to the metachromasia phenomenon, highlight the different degrees of sulfation between the two fractions, with Ht2 being the most sulfated. Through this staining protocol, non-sulfated polysaccharides are shown in blue (i.e., HMW-HA and LMW-HA), whereas the dye turns to purple (i.e., fucoidan and CS) or yellow (i.e., heparin) as the degree of sulfation increases. Standard polysaccharides: high-molecular-weight hyaluronic acid (HMW-HA), low-molecular-weight hyaluronic acid (LMW-HA), fucoidan, chondroitin sulfate (CS), and heparin (Hep).

As illustrated in Figure 1, the two AP fractions purified from *Holothuria tubulosa* consisted of a wide range of molecular weight polysaccharides. Moreover, thanks to the metachromasia phenomenon caused by the dye used, it could be appreciated that different eluted fractions exhibited different degrees of sulfation. It is evident from the color and the density that the Ht2 fraction exhibits a significantly higher degree of sulfation as compared to Ht1, as expected from the chromatographic elution order (1.0 and 0.5 M, respectively).

### 3.2. Biological Activities of AP Enriched Fractions

The two polysaccharide fractions extracted from *Holothuria tubulosa* (Ht1 for the 0.5 M fraction; Ht2 for the 1.0 M fraction) were assessed for their potential effects on in vitro viability, morphological properties, and migratory potential of MDA-MB-231 cells.

**Figure 2:**
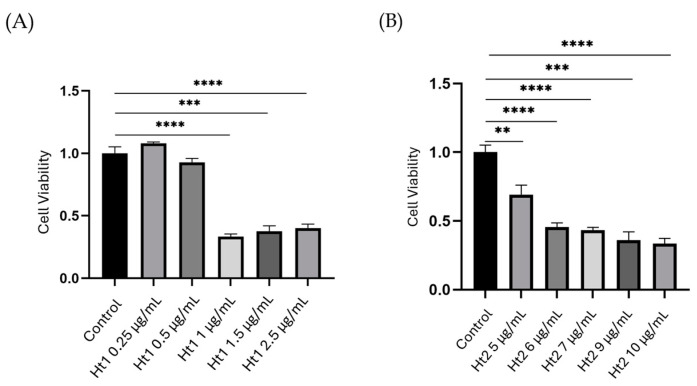
Evaluation of the effects of Ht1 and Ht2 on cell viability. MDA-MB-231 cells were treated for 24 h with increasing concentrations of Ht1 (**A**) and Ht2 (**B**), followed by a WST-1 assay, which relies on the conversion of the tetrazolium salt into formazan by mitochondrial dehydrogenases in viable cells. Formazan formation was evaluated by measuring the absorbance at 450 nm. Cell viability in the different experimental conditions is reported as the ratio with respect to the control. Each bar represents the mean ± SD values from triplicate samples. ** *p* < 0.01, *** *p* < 0.001, **** *p* < 0.0001.

To evaluate the general effects on cell viability, a wide concentration range of both Ht1 and Ht2 was initially tested through the WST-1 assay, following a 24 h treatment. Both Ht1 and Ht2 showed dose-dependent inhibitory effects on cell viability with IC_50_ of 1.22 ± 0.32 µg/mL (y = −0.349ln(x) + 0.5708; R^2^ = 0.8071) and 6.8 ± 0.26 µg/mL (y= −0.0698x + 0.9762; R^2^ = 0.9339), respectively. As shown in Figure 2, the effective concentration range for Ht1 was between 1 and 2.5 µg/mL (panel A), whereas that for Ht2 was between 2.5 and 10 µg/mL (panel B). Based on the inhibitory effects of the two AP fractions on cell viability and their cytotoxicity on MDA-MB-231 cells, the optimum concentrations for the following studies were set at 1.5 and 5.0 μg/mL for Ht1 and Ht2, respectively.

**Figure 3:**
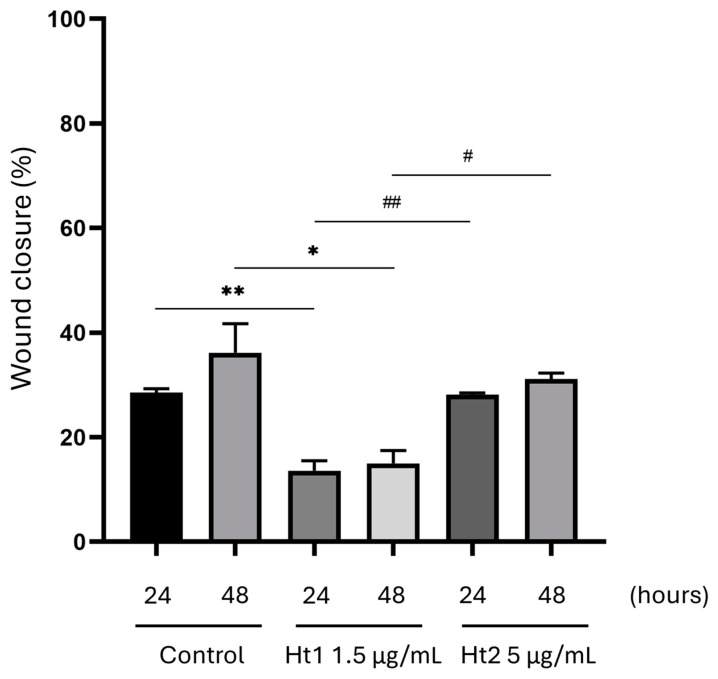
Evaluation of the effects of Ht1 and Ht2 on cell migration. The migratory potential of MDA-MB-231 cells treated with either Ht1 or Ht2, in serum-free conditions and in the presence of the cytostatic cytarabine, was evaluated by quantifying the area of the scratch with Fiji v1.52p image analysis software, following 24 h and 48 h cell culture. Each bar represents the mean ± SD values from triplicate samples. * *p* < 0.05, ** *p* < 0.01, # *p* < 0.05, ## *p* < 0.01.

Cell migration was evaluated through the wound healing assay. Briefly, a wound was created in each well by inflicting a scratch with a pipette tip on confluent cell monolayers and the migration capacity was screened after 24 h and 48 h incubation with Ht1 or Ht2. In the presence of Ht1, the migratory capacity of MDA-MB-231 cells was significantly decreased by 52% and 58%, respectively (Figure 3), suggesting that, in addition to an antiproliferative effect, this fraction also displays a significant inhibitory effect on the migration of the MDA-MB-231 cells.

**Figure 4:**
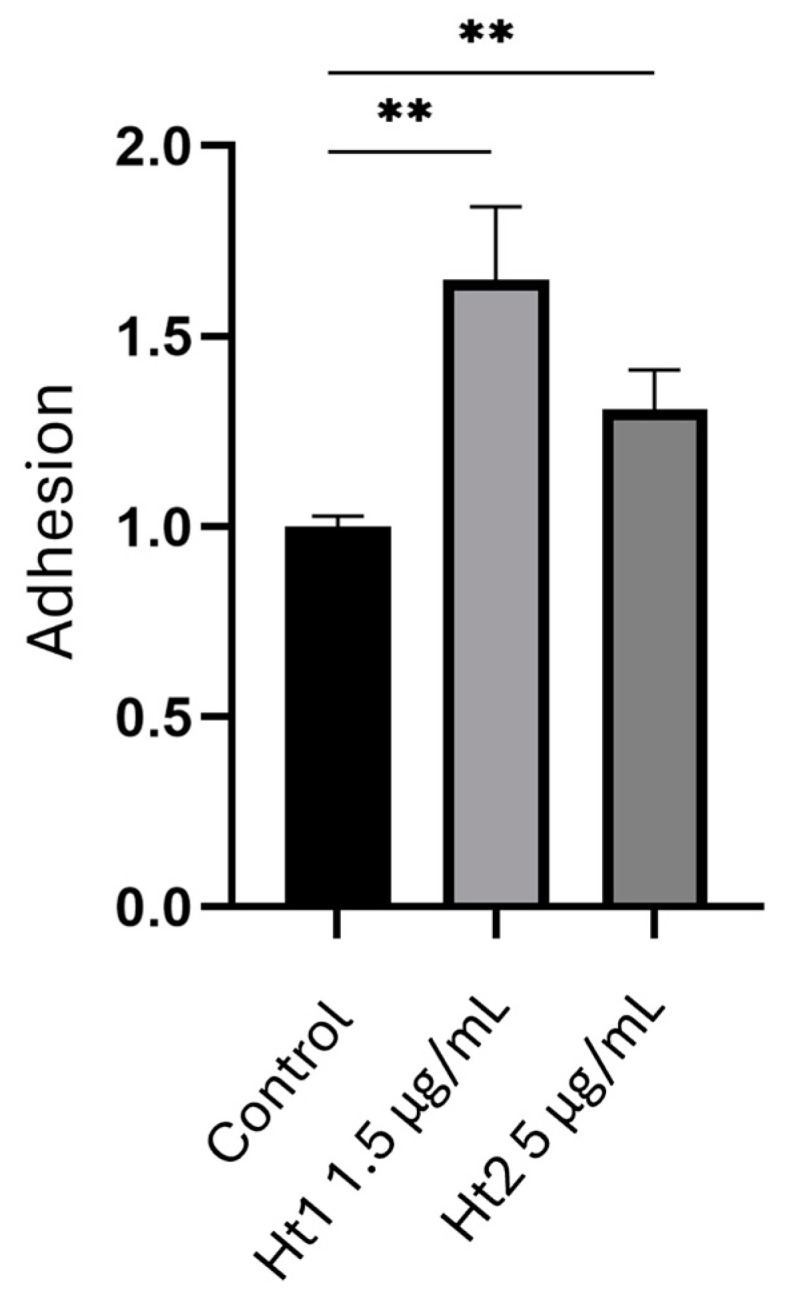
Evaluation of the effects of Ht1 and Ht2 on cell adhesion to collagen I-coated plates. Adhesive properties of MDA-MB-231 cells to collagen type I, following treatment with either Ht1 or Ht2 for 24 h, was assessed using a PrestoBlue™ assay, which relies on the reduction of resazurin reagent to resorufin by metabolically active cells. The emission at the 615 nm wavelength was assessed (excitation wavelength 535 nm) using a microplate reader. Adhesion in the different experimental conditions is reported as the ratio with respect to the control. Each bar represents the mean ± SD values from triplicate samples. ** *p* < 0.01.

Furthermore, the effects of the two AP fractions on cell adhesion were quantitatively assessed using a collagen type I adhesion assay. As illustrated in Figure 4, treatment with both Ht1 and Ht2 fractions resulted in a significant increase in the adhesion rate of the MDA-MB-231 cells, which was strongly corelated with the reduced migratory potential. This effect was more evident in the presence of the Ht1 fraction with a significant increase of adhesion on collagen I (*ca* 65%). The respective increase for the Ht2 fraction was *ca* 31% as compared to the control.

**Figure 5:**
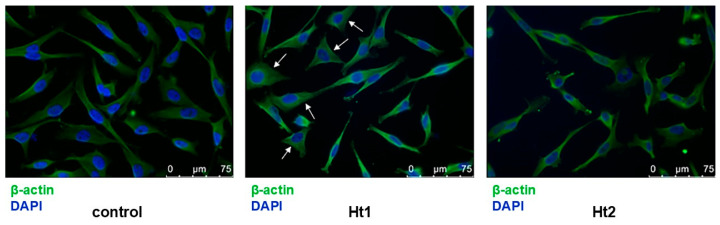
Evaluation of the effects of Ht1 and Ht2 on cytoskeleton organization. MDA-MB-231 cells were treated with either Ht1 or Ht2 for 24 h in serum-free conditions, stained with DAPI (blue) and β-actin (green), and observed using confocal microscopy. Arrows indicate the alterations toward a more epithelial-like morphology and flattened shape.

In order to evaluate the effects of the Ht1 and Ht2 fractions on cell morphology, MDA-MB-231 cells were cultured on 2D coverslips for 24 h and stained for nuclei morphology and cytoskeleton organization (β-actin). Notably, treatment with the Ht1 fraction altered the morphological characteristics of the mesenchymal (spindle-like) MDA-MB-231 cells to a more epithelial one (shown by arrows in Figure 5), suggesting a pharmacological potential for Ht1. This phenomenon was not as pronounced for the Ht2 fraction. Confocal microscopy using β-actin staining revealed notable morphological differences among the cells. Cells indicated by arrows exhibit a more epithelial-like phenotype, characterized by an expanded, flattened morphology and prominent actin organization.

**Figure 6:**
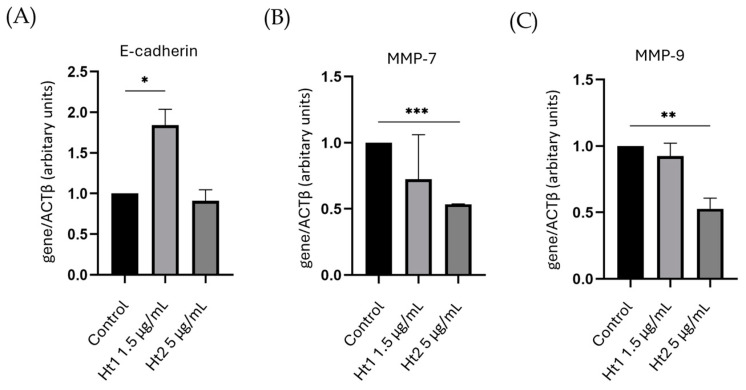
Evaluation of the effects of Ht1 and Ht2 on gene expression. RT–qPCR analysis of E-cadherin (**A**) and MMPs (panels (**B**,**C**)) expression in MDA-MB-231 cells treated with the two polysaccharide-enriched fractions relative to untreated controls. Each bar represents mean ± SD values from duplicate samples. * *p* < 0.05, ** *p* < 0.01, *** *p* < 0.001.

These findings prompted us to investigate the effects of the two AP fractions on the expression of genes implicated in the EMT/MET processes and, ultimately, on metastatic potential. After quantification and purity assessment, total RNA was reverse-transcribed into complementary DNA, and mRNA expression levels were evaluated through RT–qPCR analysis. The obtained results showed a significant increase in the expression of the epithelial marker E-cadherin following treatment with Ht1 (Figure 6A). This is in accordance with the alterations of cell morphology (Figure 5) observed upon treatment with the Ht1 fraction. For its part, the Ht2 fraction exhibited a significant suppressive effect on the expression of the MMP-7 and MMP–9 genes (Figure 6, panels B and C, respectively).

**Figure 7:**
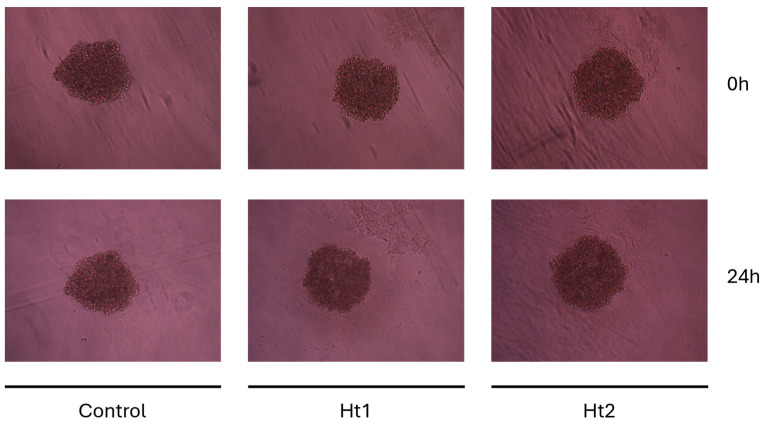
Effects of Ht1 and Ht2 AP fractions on 3D cell culture model. MDA-MB-231–derived spheroids cultured in U-shaped low adhesion 96-well plates in the presence or absence of either Ht1 or Ht2 fraction for 24 h showed no significant variations in growth or shape.

An additional assessment was conducted on MDA-MB-231 spheroids in order to evaluate the effects of the two fractions on a 3D cancer cell model. 3D cultures may provide a more accurate representation of the tumor microenvironment than traditional 2D models. To this end, MDA-MB-231-derived spheroids were treated for 24 h (Figure 7) with both Ht fractions, showing no significant effects on spheroid growth and size. This may be related to the highly hydrophilic properties and charge density of both APs, which could hinder their permeation through the spheroids. Specific delivery vehicles incorporating such APs may be used for future studies and pharmacological approaches.

## 4. Discussion

Our results have demonstrated a significant decrease in metabolic activity and migratory potential of MDA-MB-231 cells following treatment with the extracted AP fractions. This evidence suggests that APs fractions may interfere with key signaling pathways involved in cellular metabolism and growth. This aligns with previous studies demonstrating that various natural compounds from marine organisms can inhibit metabolic pathways in cancer cells, thereby reducing their viability and growth [32,33]. The significantly decreased migratory potential and the increased adhesion of MDA-MB-231 cells by Ht1 fraction suggests that this AP fraction may inhibit the invasive properties of the aggressive cancer cells, which is a critical factor in tumor metastasis [34,35]. This is further supported by our findings on cell morphological changes induced by Ht1 showing the transition of the mesenchymal-like morphology to a more epithelial one.

E-cadherin is a crucial calcium-dependent cell adhesion molecule encoded by the CDH1 gene located on chromosome 16q22.1 [36,37]. It has an important role in cell differentiation, polarity, and maintaining the integrity of epithelial cells. Furthermore, E-cadherin plays crucial roles in contact inhibition of cell proliferation and the loss of its expression is associated with increased cell motility and invasiveness in cancer. Indeed, E-cadherin acts as a tumor suppressor by limiting the ability of cancer cells to detach from the primary tumor and invade surrounding tissues [38].

Our results indicate that the observed alteration in morphological properties of MDA-MB-231 cells, treated with Ht1 is in close association with the enhanced gene expression of E-cadherin.

MMPs actively participate in the entire metastatic pathway through their biological functions, including their capacity to degrade ECM components and their ability to interact with and regulate different growth factors, cytokines, and chemokines [39]. MMP-7 directly cut off the E-cadherin ectodomain releasing an 80 kDa fragment involved in the inhibition of epithelial cell aggregation and cell invasion induction in a paracrine manner [40]; MMP9, also known as gelatinase B, plays an important role in ECM remodeling and is associated with tumor invasion, metastasis, and modulation of tumor microenvironment. Its high expression in breast cancer has been associated with reduced patient survival [41]. The observed reduction in MMP-7 and MMP-9 mRNA levels suggests that Ht2 may modulate the ECM remodeling, potentially impairing tumor cell invasion and metastasis. Overall, the potential anticancer effects of the Ht1 and Ht2 fractions may be mediated by distinct biochemical pathways, which require further investigation.

3D models mimic the in vivo tumor microenvironment more accurately than traditional 2D cultures [42], providing valuable insights into the therapeutic potential of these polysaccharides. Although only limited effects on spheroids have been evidenced, possibly due to the AP high hydrophilicity and charge density, further attempts will be performed by incorporating these polysaccharides in specific delivery vehicles.

**Figure 8:**
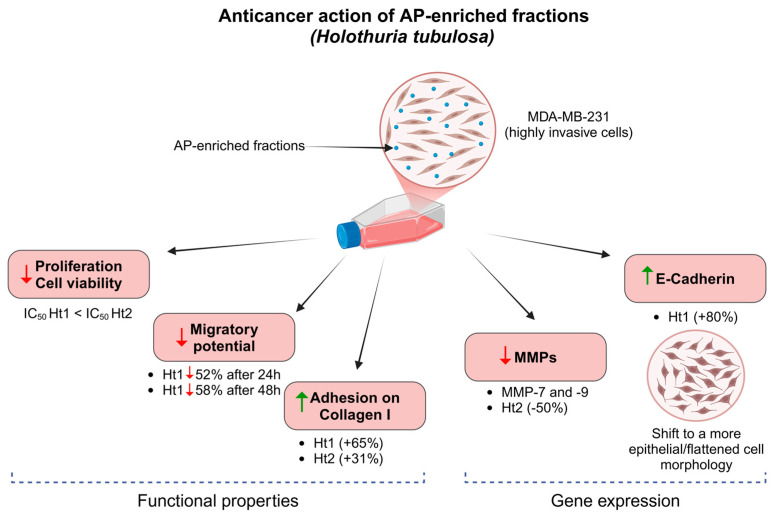
Schematic showing the main actions of *Holothuria tubulosa* AP-enriched fractions (Ht1 and Ht2) on the highly aggressive MDA-MB-231 cell line at the cellular and molecular levels. In addition to the anticancer action observed for both fractions on cancer cells’ survival, Ht1 reduced their migratory potential and both APs ameliorated their adhesive properties at different levels. The transition to a more epithelial-like flattened cell morphology associated with less aggressive properties as compared to the mesenchymal-like phenotype of untreated MDA-MB-231 cells was strongly associated with the increase of the epithelial marker E-Cadherin induced by Ht1. Ht2 also had a suppressive effect on the expression of two MMPs (MMP-7 and -9) related to metastatic potential. Created with BioRender.com.

The obtained data are summarized in Figure 8. The potential anticancer properties of APs should be further investigated in respect to their mechanisms of action. Their effects might arise from their interactions with the receptors closely associated with proteoglycans/glycosaminoglycans [43]. One possible mechanism could be the competitive action of binding of APs, which could affect cell signaling and gene expression. Additionally, binding of APs with matrix effectors, such as matrix degrading enzymes and growth factors, could potentially affect the cell-matrix interactions and cell functional properties such as proliferation, migration, and adhesion.

## 5. Conclusions

The vast class Holothuroidea represents a crucial renewable source of many bioactive compounds, valuable for applications such as pharmacology and regenerative medicine [2,5].

This preliminary study provides evidence that the acidic polysaccharides extracted from *Holothuria tubulosa* exert significant inhibitory effects on both the proliferative and migratory potential of a well-characterized TNBC cell line, while also modulating the expression of key ECM molecules. The exact mechanisms of action of the Ht1 and Ht2 fractions and the metabolic pathways involved have not yet been investigated but deserve to be elucidated along with the structural features of these polysaccharides, which are probably responsible for their effects. Overall, our innovative findings provide new opportunities for advancing research on targeted breast cancer therapeutics and strongly suggest these marine-derived polysaccharides are promising molecules for the development of novel therapeutic strategies.

## Figures and Tables

**Table 1:** Primer sequences of the genes of interest for RT–qPCR analysis.

Gene	Forward Primer	Reverse Primer
ACTβ	5′-TCAAGATCATTGCTCCTCCTGAG-3′	5′-ACATCTGCTGGAAGGTGGACA-3′
CDH1 (E-cadherin)	5′-TACGCCTGGGACTCCACCTA-3′	5′-CCAGAAACGGAGGCCTGAT-3′
MMP7	5′-GCTGGCTCATGCCTTTGC-3′	5′-TCCTCATCGAAGTGAGCATCTC-3′
MMP9	5′-TTCCAGTACCGAGAGAAAGCCTAT-3′	5′-GGTCACGTAGCCCACTTGGT-3′

## Data Availability

The original contributions presented in this study are included in the article. Further inquiries can be directed to the corresponding author.

## Abbreviations

The following abbreviations are used in this manuscript:

AP	acidic polysaccharide
Ht	*Holothuria tubulosa*
Ht1	polysaccharide-enriched fraction 1 eluted with 20 mM Tris–HCl buffer, pH 8.6, containing 0.5 M lithium chloride
Ht2	polysaccharide-enriched fraction 2 eluted with 20 mM Tris–HCl buffer, pH 8.6, containing 1 M lithium chloride
EMT	epithelial–mesenchymal transition
ER	estrogen receptor
HER2	human epidermal growth factor receptor 2
PR	progesterone receptor
TNBC	triple-negative breast cancer
MDA-MB-231	triple-negative breast cancer cell line
ECM	extracellular matrix
MMP	matrix metalloproteinases
FCS	fucosylated chondroitin sulfate
DDT	dehydrated and delipidated tissue
DEAE	diethylaminoethyl
UA	uronic acid
C-PAGE	carbohydrate polyacrylamide gel electrophoresis
WST-1	water-soluble tetrazolium salt
